# The Stereoselectivity and Hydrolysis Efficiency of Recombinant d-Hydantoinase from *Vigna angularis* Against 5-Benzylhydantoin Derivatives with Halogen and Methyl Substituents

**DOI:** 10.1007/s12010-014-1313-4

**Published:** 2014-10-24

**Authors:** Gniewomir Latacz, Katarzyna Kieć-Kononowicz

**Affiliations:** Department of Technology and Biotechnology of Drugs, Jagiellonian University Medical College, Medyczna 9, 30-688 Kraków, Poland

**Keywords:** d-hydantoinase, d-amino acids, d-phenylalanine, Capillary electrophoresis

## Abstract

The researches on d-hydantoinase activity and substrate specificity towards dihydropyrimidine and hydantoin derivatives have been carried out intensively over the last few decades. So far, the major efforts have focused on (*R*,*S*)-5-phenylhydantoin and (*R*,*S*)-5-(4-hydroxyphenyl)hydantoin, the most desirable d-hydantoinase substrates from pharmaceutical industry point of view. However, it was shown that d-hydantoinase is a substrate-dependent enzyme, and its activity and stereoselectivity towards 5-monosubstituted hydantoins varied significantly with the type of substrate and the source of the enzyme. The aim of this study was to estimate the substrate specificity of d-hydantoinase towards series of 5-benzylhydantoin derivatives with halogen and methyl substituents in the phenyl ring. The biotransformations were carried out by using commercial enzyme: immobilized, recombinant, cloned, and expressed in *Escherichia coli* d-hydantoinase from *Vigna angularis* (*r*D-HYD). All reactions were monitored by capillary electrophoresis (CE), and the conversion yields were calculated. Additionally, enantiomeric ratios of the obtained d-phenylalanine derivatives were estimated by chiral high-performance liquid chromatography (HPLC). Interestingly, the differences in the activities of examined enzyme towards particular 5-benzylhydantoin derivatives were observed. CE was also shown as a promising method for monitoring the hydrolysis of new substrates by d-hydantoinase and further analyzing of enzyme substrate specificity.

## Introduction

Dihidropyrimidinase (EC 3.5.2.2) is a very important enzyme belonging to the cyclic amidohydrolases superfamily which take part in either pyrimidines or purines catabolism in bacteria, yeasts, plants, and animals. Dihidropyrimidinase catalyzes the reversible hydrolysis of 5,6-dihydrouracil as well as the alternative substrates like hydantoin and dihydrothymine [1, 2]. This enzyme, also known as hydantoinase, is commonly used in hydantoinase method for obtaining optically pure d- or l-amino acids starting from racemic 5-monosubstituted hydantoins. In this method, the substrates are hydrolyzed stereoselectively into corresponding intermediates d- or l-*N*-carbamoyl-amino acids, which can be further converted to the amino acids, either chemically with NaNO_2_/HCl or enzymatically with carbamoylase [3, 4]. The hydantoinase process has been successfully used for years in pharmaceutical industry for biosynthesis of d-phenylglycine and d-4-hydroxyphenylglicyne starting from (*R*,*S*)-5-phenylhydantoin and (*R*,*S*)-4-hydroxyphenylhydantoin [5]. These two d-amino acids are used to produce semisynthetic β-lactam antibiotics such as ampicillin or amoxicillin in the reaction of condensation with 6-aminopenicillanic acid (6-AP). From a pharmaceutical point of view, d-amino acids are also important as the chiral building blocks of new peptidomimetics. One of the preferred d-amino acids to design and synthesis of new peptidomimetics is d-phenylalanine (d-phe). The hydrophobic activity of d-phe may be responsible for the increased hydrophobicity of peptidomimetics and may result in better transport properties through cellular membranes [6]. Additionally, the peptidomimetics containing d-amino acids are resistant to proteases, peptidases and may by less immunogenic [7, 8]. d-phenylalanine and its derivatives are the building blocks of many peptidomimetics which have entered the drug market or are under clinical trials. For instance, d-phe, 4-amino-d-phe, and 4-chloro-d-phe are the components of following analogs of GnRH hormone: abarelix, acyline, antarelix, azaline b, antide, cetrorelix, degarelix, ganirelix, iturelix, ornirelix, and Nal-Glu [9, 10]. d-phe was also used for the synthesis of the analgesic opioid peptide Ac-rfwink-NH2 [11], and nateglinide—drug stimulating the release of insulin [12]. Another important peptidomimetics containing d-phe are the analogs of somatostatin hormone, such as synthetic somatostatin analogs: octreotide and vapreotide [13, 14] or heksarelin, a somatostatin-releasing stimulator [15]. d-phe was also found in the structure of peptides with strong antibacterial activity isolated from *Bacillus* sp.: gramicidin S, tyrocidine A–D [16], bacitracin A [17], and polymyxin B [18]. In view of increasing importance of d-phe in the design of new peptidomimetics, the hydantoinase method was successfully applied for the synthesis of the series of halogen and methyl d-phe derivatives. Taking into account the substrate-dependent properties of d-hydantoinases, the d-hydantoinase from *Vigna angularis*—immobilized, recombinant, and expressed in *Escherichia coli*—was used to estimate its substrate specificity towards abovementioned 5-benzylhydantoin derivatives.

## Materials and Methods

### Chemicals

Unless otherwise stated, hydantoin, benzaldehyde derivatives, red phosphorus, sodium acetate, ammonium acetate, and solvents which were used in chemical syntheses of 5-benzylhydantoin derivatives were obtained from commercial suppliers and used without further purification. Chemical syntheses were carried out as it was described previously [19].

### Biotransformations

**Fig. 1:**
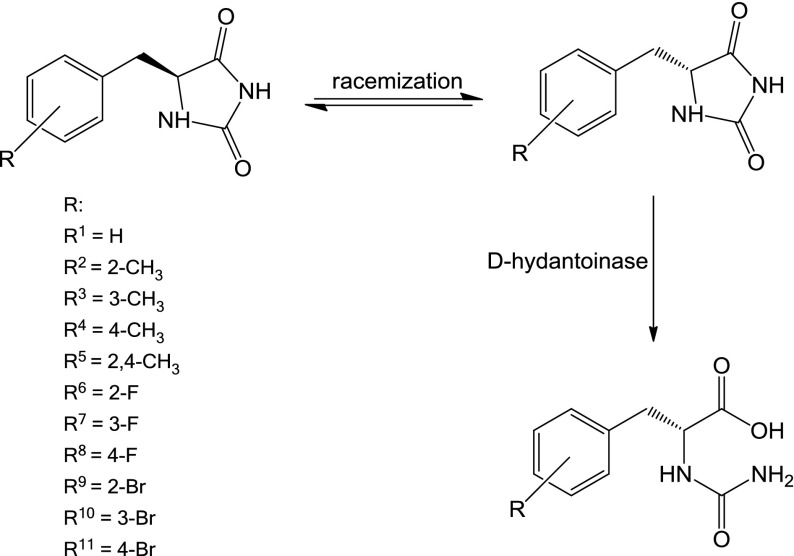
The production of *N*-carbamoyl-d-phe derivatives using *r*D-HYD

**Fig. 2:**
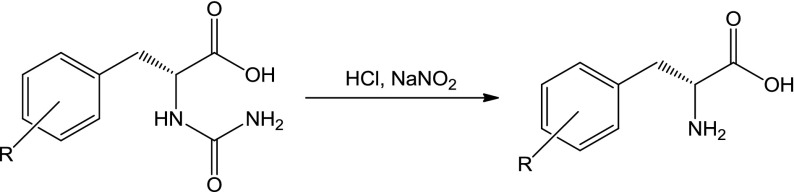
The production of d-phe derivatives from *N*-carbamoyl-d-phe using the reaction of diazotization

d-hydantoinase from *V. angularis*—immobilized, recombinant, cloned, and expressed in *E. coli* (53.1 U/g using hydantoin as a substrate)—was obtained from Sigma-Aldrich. The (*R*,*S*)-5-benzylhydantoin derivatives were first dissolved in 100 μl of dimethyl sulfoxide (DMSO) and, next, the borate buffer pH 8.9 (MERCK) up to the concentration of 1 mM was added. All biotransformations were carried out with 10 mg of examined d-hydantoinase suspended in a 2-ml solution of 1 mM 5-benzylhydantoin derivative in borate buffer pH 8.9 on the orbital shaker 120 rpm at 37 °C for 48 or 72 h (Fig. 1). The d-hydantoinase was next removed using 0.2-μm membrane filter (Whatman) and the obtained *N*-carbamoyl-d-phe derivatives were converted into the corresponding d-phe derivatives using a diazotization reaction. For this purpose, the reaction mixture was acidified first with 3.5 M HCl to pH 1.0 and next 5 μl of 3.5 M NaNO_2_ was added. Then, the reaction mixture was stored on ice for 6 h. The reaction was stopped by alkalization to pH 9.0 with 3.5 M NaOH (Fig. 2).

### Analysis

**Fig. 3:**
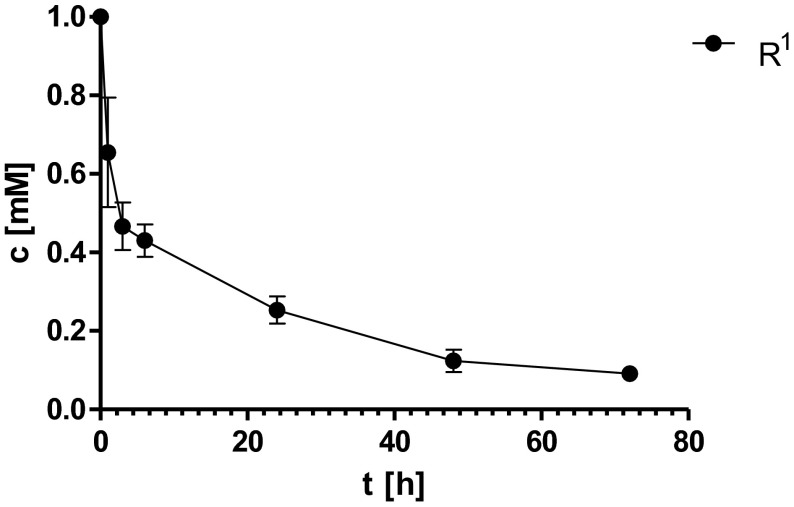
The bioconversion of (*R*,*S*)-5-benzylhydantoin (R^1^) with calculated standard deviations from three independent experiments

To calculate the calibration curves, the following concentrations of each substrate were used: 1, 0.7, 0.5, 0.1, and 0.02 mM. The calibration curves were estimated by Beckman capillary electrophoresis (CE) system (P/ACE MDQ) controlled by 32 Karat Software version 8.0 and equipped with diode-array detector (DAD). An uncoated fused-silica capillary with total length of 60 cm (50.2 cm to detection window) and internal diameter 75 μm was also purchased from Beckman. DMSO was used as an internal standard and electroosmotic flow (EOF) marker for all separations. All analyses by CE were performed at room temperature, applying a voltage of 20 kV and at pH 8.9 of the background electrolyte. The time-course curves of reaction were followed by CE determination of concentration of the respective (*R*,*S*)-5-benzylhydantoin derivative. Data were collected after 6, 24, and 48 h and additionally, if total conversion was not observed, after 72 h of the reaction. The experiments were performed once. To demonstrate the reproducibility, the reactions for the one substrate (R^1^) were conducted in three repetitions. Data were collected after 1, 3, 6, 24, 48, and 72 h and the standard deviations from three independent experiments were calculated (Fig 3). Enantiomeric excess (ee) of the obtained d-phe derivatives was measured using HPLC Dionex P580 instrument equipped with a ChiroSil (RCA+) column containing the chiral stationary phase prepared by a covalent trifunctional bonding of (+) or (−)-(18-crown-6)-tetracarboxylic acid. The mobile phase was prepared according to the manufacturer’s instructions dedicated for the enantioseparation of d,l-phenylalanine and was composed of 70 % methanol/30 % 10 mM CH_3_COOH. The flow rate was 1.5 ml/min, the temperature 25 °C, and the detection wavelength 225 nm.

## Results and Discussion

**Fig. 4:**
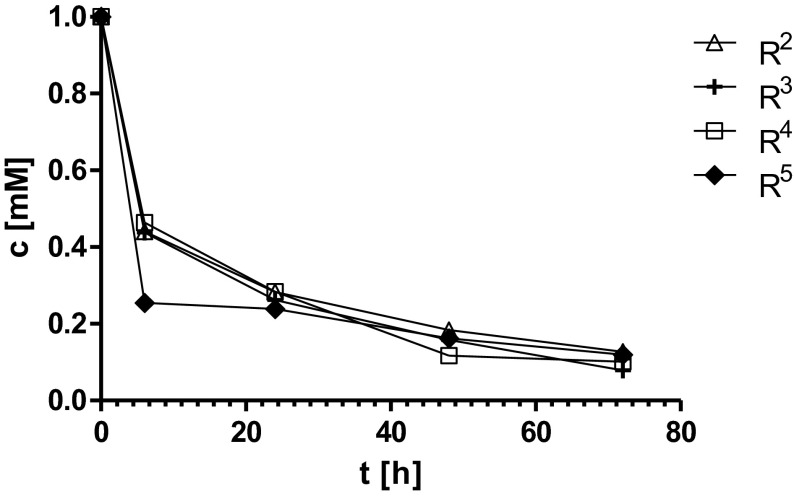
The bioconversion of (*R*,*S*)-5-benzylhydantoin derivatives R^2^–R^5^ with *r*D-HYD in sodium borate buffer pH 8.9 at 37 °C for 72 h

**Fig. 5:**
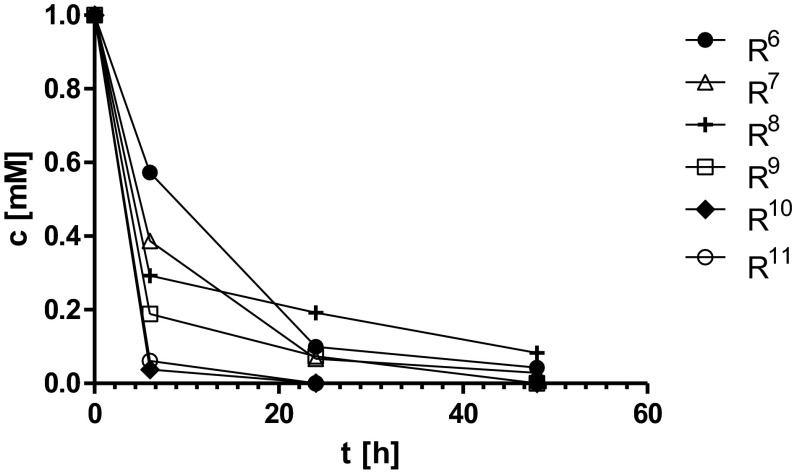
The bioconversion of (*R*,*S*)-5-benzylhydantoin derivatives R^6^–R^11^ with *r*D-HYD in sodium borate buffer pH 8.9 at 37 °C for 48 h

There are many examples in the literature describing the ability of d-hydantoinase to hydrolyze the broad range of substrates. However, the activity of this enzyme as well as enantioselectivity was proven to be substrate-dependent. For instance, the d-hydantoinase obtained from adzuki bean (*V. angularis*) was shown to be 5.3–7 fold more active against (*R*,*S*)-phenylhydantoin, than against (*R*,*S*)-4-hydroxyphenylhydantoin [20]. Another important example was the enantioseletivity of d-hydantoinase from *Arthrobacter aurescens* DSM 3745, which differed according to the used substrate. The biotransformation catalyzed by this enzyme was l-selective for indolylmethylhydantoin, non-selective for methylthioethylhydantoin (with slight preference for the d-enantiomer), and d-selective for methylhydantoin [21]. Several bacterial strains with d-hydantoinase activity and additionally three recombinant d-hydantoinases were shown to be able to produce *N*-carbamoyl-β-phenylalanine from racemic 6-phenyl-5,6-dihydrouracil. However, the enantiomeric excess of the obtained d-enantiomer depended on biocatalyst and was from 6 to 96 %, while one reaction resulted surprisingly in 61 % of enaniomeric excess of l-enantiomer. Additionally, the conversion efficiency of these reactions varied from 12 to 85 % [22]. Moreover, our recent studies on (*R*,*S*)-5-benzylhydantoin derivatives biotransformation by d-hydantoinase showed differences in bioconversion efficiency and enantioselectivity with the preference to the substrates possessing the substituent in the 4-position of phenyl ring [23]. The docking studies and the study on modification of the enzyme’ s hydrophobic-binding pocket by mutagenesis showed also the correlation between the size of the substrate, its spatial orientation, and accessing to the catalytic center of d-hydantoinase [24, 25]. During this study, we used capillary electrophoresis as a tool for monitoring the progress of the substrate hydrolysis and to calculate the reaction efficiency catalyzed by *r*D-HYD. Additionally, the chiral high-performance liquid chromatography (HPLC) technique was applied to determine the enantiomeric ratios of obtained d-phenylalanine derivatives. As it was presented in Table 1, all substrates were successfully converted into *N*-carbamoyl-d-amino acids with very high bioconversion efficiency from 87.32 to 100 %. However, we observed the greater preference of the examined enzyme rather to the substrates with halogen (R^6^–R^11^) than with methyl (R^2^–R^5^) substituents. All fluorine- and bromine-substituted substrates were converted with almost 100 % efficiency after 48 h of incubation, whereas none of methyl-substituted substrates was completely hydrolyzed after 72 h (Figs. 4 and 5). Moreover, all the bromine-substituted (R^9^–R^11^) derivatives were converted with more than 80 % efficiency as early as 6 h of incubation (Fig. 5). The comparison of enantiomeric ratios of the obtained d-phenylalanine derivatives showed that the high conversion efficiency of halogen derivatives by *r*D-HYD was not always affected on its enantioselectivity. For instance, despite of the almost 100 % bioconversion efficiency of 2-fluorine benzylhydantoin derivative R^6^, the enantiomeric excess of obtained 2-fluorine-d-phenylalanine was the lowest among all examined products and resulted only 77.30 %. However, all bromine d-phenylalanine derivatives (R^9^–R^11^) were obtained with high enantiomeric purity from 92.53 to 94.33 % as well as with the highest conversion efficiency among all examined reactions, indicating distinctly the substrate preferences of *r*D-HYD (Table 1). Interestingly, the noticed previous preference of that enzyme to (*R*,*S*)-5-benzylhydantoin derivatives with substituent in 4-position [23] in case of methyl and halogen derivatives was not observed. Thus, further docking analyses and the study on the potential influence of (*R*,*S*)-5-benzylhydantoin derivatives racemization or product inhibition on *r*D-HYD stereoselectivity and reaction efficiency should be performed.

**Table 1:** Biotransformations efficiency and enantioselectivity

R	ee (%)	Conversion (%)
R^1^	95.51	89.97
R^2^	78.86	87.32
R^3^	92.76	92.13
R^4^	79.18	89.95
R^5^	97.82	88.06
R^6^	77.30	99.53^a^
R^7^	87.58	99.68^a^
R^8^	95.73	97.36^a^
R^9^	92.53	100^a^
R^10^	93.38	100^a^
R^11^	94.33	100^a^

*R* substrate, *ee* enantiomeric excess

^a^Conversion after 48 h
